# Genome-wide systematic characterization of the *HAK/KUP/KT* gene family and its expression profile during plant growth and in response to low-K^+^ stress in *Saccharum*

**DOI:** 10.1186/s12870-019-2227-7

**Published:** 2020-01-13

**Authors:** Xiaomin Feng, Yongjun Wang, Nannan Zhang, Zilin Wu, Qiaoying Zeng, Jiayun Wu, Xiaobin Wu, Lei Wang, Jisen Zhang, Yongwen Qi

**Affiliations:** 1 Guangdong Key Lab of Sugarcane Improvement & Biorefinery, Guangdong Bioengineering Institute (Guangzhou Sugarcane Industry Research Institute), Guangzhou, 510316 China; 2 Guangzhou Guansheng Breeding Research Institute, Guangzhou, 511453 China; 3 Center for Genomics and Biotechnology, Fujian Provincial Key Laboratory of Haixia Applied Plant Systems Biology, College of Crop Science, Fujian Agriculture and Forestry University, Fuzhou, 350002 China

**Keywords:** *Saccharum*, *HAK/KUP/KT*, Evolution, Gene expression, Low-K^+^ stress

## Abstract

**Background:**

Plant genomes contain a large number of HAK/KUP/KT transporters, which play important roles in potassium uptake and translocation, osmotic potential regulation, salt tolerance, root morphogenesis and plant development. Potassium deficiency in the soil of a sugarcane planting area is serious. However, the *HAK/KUP/KT* gene family remains to be characterized in sugarcane (*Saccharum*).

**Results:**

In this study, 30 *HAK/KUP/KT* genes were identified in *Saccharum spontaneum*. Phylogenetics, duplication events, gene structures and expression patterns were analyzed. Phylogenetic analysis of the *HAK/KUP/KT* genes from 15 representative plants showed that this gene family is divided into four groups (clades I-IV). Both ancient whole-genome duplication (WGD) and recent gene duplication contributed to the expansion of the *HAK/KUP/KT* gene family. Nonsynonymous to synonymous substitution ratio (Ka/Ks) analysis showed that purifying selection was the main force driving the evolution of *HAK/KUP/KT* genes. The divergence time of the *HAK/KUP/KT* gene family was estimated to range from 134.8 to 233.7 Mya based on Ks analysis, suggesting that it is an ancient gene family in plants. Gene structure analysis showed that the *HAK/KUP/KT* genes were accompanied by intron gain/loss in the process of evolution. RNA-seq data analysis demonstrated that the *HAK/KUP/KT* genes from clades II and III were mainly constitutively expressed in various tissues, while most genes from clades I and IV had no or very low expression in the tested tissues at different developmental stages. The expression of *SsHAK1* and *SsHAK21* was upregulated in response to low-K^+^ stress. Yeast functional complementation analysis revealed that *SsHAK1* and *SsHAK21* could rescue K^+^ uptake in a yeast mutant.

**Conclusions:**

This study provided insights into the evolutionary history of *HAK/KUP/KT* genes. *HAK7/9/18* were mainly expressed in the upper photosynthetic zone and mature zone of the stem. *HAK7/9/18/25* were regulated by sunlight. *SsHAK1* and *SsHAK21* played important roles in mediating potassium acquisition under limited K^+^ supply. Our results provide valuable information and key candidate genes for further studies on the function of *HAK/KUP/KT* genes in *Saccharum*.

## Background

Potassium is an essential mineral nutrient for plant growth and development and is also the most abundant monovalent cation in plants, accounting for approximately 2~10% of plant dry weight [1]. Potassium is involved in many important physiological and biochemical processes, such as cell turgor regulation, cell charge balance regulation, enzyme activity regulation and protein synthesis [1]. Symptoms of plant potassium deficiency usually manifest as weak stems, easy lodging, decreased tolerance to drought and cold and yellow leaves, due to the degradation of proteins and chlorophyll, which leads to tissue necrosis [2]. Thus, potassium is of great importance for improving crop yield and quality. Sugarcane is an important sugar and energy crop with a long growth period, large biomass and large amount of potassium fertilizer absorption. On the one hand, it is estimated that sugarcane needs to absorb approximately 2~2.5 kg of potassium to produce one ton of sugar [3, 4]. On the other hand, sugarcane is mainly cultivated in subtropical and tropical regions, where soil acidification and potassium leaching are common. The contents of total potassium and available potassium in the cultivated layer of these sugarcane areas are low.

Plant cells maintain a relatively high and stable K^+^ concentration (approximately 100~150 mM) in the cytosol, while the K^+^ concentration is highly variable in the range of 0.01~1 mM [5]. It is generally believed that there are two mechanisms for potassium uptake by plants, namely, a high-affinity transport system (HATS) via potassium transporters at low external potassium concentrations (< 0.2 mM) and a low-affinity transport system (LATS) via potassium channels at high potassium concentrations (> 0.5 mM) [6, 7]. According to their structure and function, potassium transporters in plants can be divided into five families: (1) Shaker channels; (2) TPK (tandem-pore K^+^) channels; (3) HAK (high-affinity K^+^ transporter)/KUP (K^+^ uptake permease)/KT (K^+^ transporter); (4) HKT transporters; and (5) CPAs (cation-proton antiporters) [2, 8]. Among them, the *HAK/KUP/KT* family is the largest and is widely distributed in bacteria, fungi and plants but has not been identified in animal cells [9].

According to their homology with bacterial KUP and fungal HAK transporters [10], the plant HAK/KUP/KT transporter members *AtKUP1* and *HvHAK1* were first cloned from Arabidopsis and barley [11, 12]. Both genes could complement K^+^ uptake-deficient strains of yeast, indicating that they had potassium transporter activity. Subsequently, several *HAK/KUP/KT* members were cloned and identified, such as *AtKUP3* and *AtHAK5* in Arabidopsis, *OsHAK1* in rice and *CaHAK1* in pepper, which were also shown to be highly compatible potassium transporters [13–16]. Based on comparative genomic methods, 13, 27 and 27 *HAK/KUP/KT* genes were identified in Arabidopsis, rice and maize, respectively [17–19]. These predicted HAK/KUP/KT transporters were sorted into four clusters. HAK/KUP/KT K^+^ transporters play versatile roles in potassium ion acquisition and transport, salt stress, osmotic regulation, and the morphogenesis of root and phenotype in plants [7]. The expression of *OsHAK1* was greatly induced in the roots of K^+^-starved rice, while *OsHAK5* was less expressed in roots but abundantly expressed in shoots [20, 21]. Some ions, particularly Na^+^ and NH_4_^+^, can have additional effects on the expression of *HAK/KUP/KT* genes [22, 23].

The transcriptional regulation of HAK/KUP/KT K^+^ transporters is a universal mechanism by which different plant species respond to K^+^-starvation stress [8]. The *HAK/KUP/KT* genes in clade I, such as *AtHAK5*, *OsHAK1*, *CaHAK1* and *ThHAK5*, display low expression levels both in roots and shoots under control conditions and are highly upregulated in roots upon K^+^-deficiency stress [12–14, 16]. While the HAK/KUP/KT K^+^ transporters in other three clades exhibit different expression patterns [24], since transcription of most K^+^ transporters are not induced by K^+^ starvation [25]. In Arabidopsis, several transcription factors, including bHLH121 (basic helix-loop-helix 121), DDF2 (dwarf and delayed flowering 2), JLO (jagged lateral organs) and TFII_A (transcription initiation factor II_A gamma chain), have been identified to bind the promoters of *HAK5* and activate its expression under low K^+^ stress [26]. Activation of HAK/KUP/KT K^+^ transporters is also regulated at posttranscriptional and/or posttranslational level. *AtHAK5* and its homologs from pepper and tomato can be activated by the CIPK23 (CBL-interacting protein kinase 23)/CBL (calcineurin B-like protein) complex [27].

In summary, numerous studies have been performed in the functional research of plant HAK/KUP/KT potassium transporters, and important progress has been made. However, the known functional *HAK/KUP/KT* genes have mainly been identified in a few plants, such as Arabidopsis, rice and maize, but their physiological functions and regulatory mechanisms in sugarcane remain unknown. In this study, based on the newly released *S. spontaneum* genome [28], we identified the *HAK/KUP/KT* gene family in *S. spontaneum*. Phylogenetic relationships among different species, exon/intron organization and gene expression were analyzed. Altogether, these results provide valuable information and robust candidate genes for future functional analyses for the genetic improvement of potassium-utilization efficiency in sugarcane.

## Results

### Identification of *HAK* genes in sugarcane

Based on comparative genomics, 29 *SbHAK* genes were identified from sorghum (*Sorghum bicolor*, sugarcane’s nearest relative). Using the protein sequences of sorghum *HAK* genes as a reference, 30 distinct *S. spontaneum HAK* genes (Table 1), excluding alleles, were identified from the genome of tetraploid *S. spontaneum* AP85–441 [28]. Each of these genes contained one to four alleles, with an average of 3 (Additional file 1). The 30 *SsHAK* genes were distributed on seven *S. spontaneum* chromosomes: chromosome 1 contained six genes; chromosome 2 contained seven genes; chromosome 3 contained four genes; chromosome 4 contained two genes; chromosome 5 contained five genes; and chromosome 6 and 8 each contained three genes. No *SsHAK* genes were identified on chromosome 7 (Additional file 1).

**Table 1:** Overview and comparison of *HAK* genes in *Saccharum spontaneum* and *Sorghum bicolor*

*Sorghum bicolor*						*Saccharum spontaneum*						Similarity^f^
Gene	AA^a^	pI^b^	Mw^c^ (kDa)	TMS^d^	P.L.^e^	Gene	AA^a^	pI^b^	Mw^c^ (kDa)	TMS^d^	P.L.^e^	
Sobic.006G061300	788	8.75	87.13	12	PM	*SsHAK1*	780	8.83	86.84	12	PM	94.42%
Sobic.003G418100	783	8.91	87.53	12	PM	*SsHAK2*	788	8.85	88.18	12	PM	94.61%
Sobic.003G164400	811	8.4	89.60	10	PM/ER	*SsHAK3*	785	8.69	86.79	11	PM	97.34%
Sobic.007G153001	706	8.37	78.02	9	PM/ER	*SsHAK4*	702	8.90	78.08	9	PM	92.92%
Sobic.003G413600	775	8.78	86.36	11	PM	*SsHAK5a*	705	8.39	78.76	11	PM	85.64%
Sobic.003G413700	775	8.54	86.42	11	PM	*SsHAK5b*	750	7.58	83.86	10	PM	93.35%
Sobic.002G411500	788	8.8	87.72	13	PM	*SsHAK7*	818	8.81	91.32	13	PM/Vacu	90.95%
Sobic.001G379900	805	7.36	89.80	12	PM/Cyto	*SsHAK8*	770	8.36	85.88	11	PM/ER	93.18%
Sobic.002G417500	792	6.96	87.53	12	PM/Cyto	*SsHAK9*	743	8.39	82.35	11	PM/ER	91.34%
Sobic.010G197500	820	8.37	91.15	10	PM/ER	*SsHAK10*	755	8.94	83.57	10	PM/Vacu	90.52%
Sobic.006G213500	805	8.33	89.66	13	PM/ER	*SsHAK11*	719	7.24	80.33	12	PM/ER	92.06%
Sobic.007G075100	790	8.21	88.50	14	PM	*SsHAK12*	509	8.54	57.87	8	PM	87.93%
Sobic.010G224400	779	8.97	85.92	12	PM/Cyto	*SsHAK13*	757	8.62	83.38	12	PM/ER	95.76%
Sobic.002G313900	843	5.71	93.38	12	PM/ER	*SsHAK14*	811	5.88	90.03	11	PM	91.12%
Sobic.006G210700	743	8.85	82.93	12	PM/ER	*SsHAK15*	852	6.00	95.04	12	PM/ER	90.12%
Sobic.001G184000	817	8.91	92.60	12	PM	*SsHAK16a*	487	9.26	55.84	8	PM/Cyto	81.06%
Sobic.001G184100	810	8.61	91.65	11	PM/ER	*SsHAK16b*	802	8.69	91.07	12	PM/ER	96.03%
Sobic.002G220600	708	8.77	78.15	12	PM	*SsHAK17*	701	9.06	78.01	12	PM	93.57%
Sobic.002G130800	787	8.69	88.61	14	PM/ER	*SsHAK18*	788	8.35	88.56	14	PM/ER	96.45%
Sobic.006G062100	746	7.29	83.31	12	PM/Golgi	*SsHAK19a*	767	7.00	85.62	10	PM/Golgi	94.78%
Sobic.006G062100	746	7.29	83.31	12	PM/Golgi	*SsHAK19b*	730	6.65	81.30	9	PM/Vacu	93.33%
Sobic.004G160000	735	8.46	80.43	12	PM/ER	*SsHAK20a*	730	8.81	80.09	12	PM/ER	97.01%
Sobic.006G061700	788	8.66	88.27	11	PM/Cyto	*SsHAK20b*	794	8.60	89.03	11	PM/Golgi	83.01%
Sobic.001G183700	828	8.51	92.29	11	PM/Cyto	*SsHAK21*	818	8.22	91.50	11	PM/ER	95.17%
Sobic.002G001800	931	8.61	102.07	12	PM/Chlo	*SsHAK22*	967	9.08	106.49	11	PM/Vacu	88.52%
Sobic.002G188600	852	6.78	93.82	12	PM/ER	*SsHAK23*	846	6.55	93.13	12	PM	98.00%
Sobic.010G112800	773	8.39	85.44	12	PM/Chlo	*SsHAK24*	698	7.62	77.44	10	PM/Chlo	96.94%
Sobic.004G250700	774	7.34	86.29	13	PM/ER	*SsHAK25*	800	7.13	89.27	14	PM/ER	94.62%
Sobic.007G209900	774	9.08	82.47	10	PM/Chlo	*SsHAK26*	744	8.98	82.93	10	PM/Chlo	89.63%
Sobic.001G184300	814	8.32	91.82	11	PM/ER	*SsHAK27*	812	8.44	91.41	11	PM/ER	97.67%

*PM* Plasma membrane, *ER* Endoplasmic reticulum, *Vacu* Vacuole, *Cyto* Cytoplasm, *Golgi* Golgi body, *Chlo* Chloroplast

^a^ Amino acid number in HAK protein sequences

^b^ Isoelectric point (pI) predicted by ExPASy (https://web.expasy.org/compute_pi/)

^c^ Molecular weight (Mw) predicted by ExPASy (https://web.expasy.org/compute_pi/)

^d^ Number of transmembrane domains possessed by HAKs, as predicted by TMHHM Server v.2.0 (http://www.cbs.dtu.dk/ services/TMHMM/)

^e^ Subcellular location of the HAK proteins predicted by WoLF PSORT (https://www.genscript.com/wolf-psort.html)

^f^ Protein sequence similarity between sorghum and sugarcane calculated by BLASTP

All 30 predicted SsHAK proteins had a typical “K_trans” domain (PF02705), which is specific to HAK/KUP/KT potassium transporter family members. For consistency, these *SsHAK* genes were named based on the previously reported *O. sativa HAK* nomenclature and phylogenetic relationships [17]. If two *SsHAK* genes were equally close to a single *OsHAK* gene, then the same name was used, followed by the letters “a” and “b” (Table 1). Two paralogous *SsHAK* genes (*SsHAK19a* and *SsHAK19b*) were identified that corresponded to the same sorghum gene, Sobic.006G062100, which may be caused by gene loss in sorghum or gene duplication in sugarcane. The number of amino acids in the 30 identified SsHAKs ranged from 487 to 967, with an average of 758. The predicted isoelectric points (pI) of the SsHAKs varied from 5.88 to 9.26, and the average pI was 8.15. The molecular weight ranged from 55.84 kDa to 106.49 kDa, with an average of 84.47 kDa (Table 1). The prediction of transmembrane domains in the SsHAK proteins indicated that most contained 11 or 12 transmembrane helices, which was similar to the findings in sorghum. The subcellular locations of the SsHAK proteins predicted by WoLF PSORT were mainly the plasma membrane, which is most suitable for their roles as transporters to maintain K^+^ homeostasis in sugarcane. In addition, the SsHAK proteins were also located on some organelles, including the endoplasmic reticulum, vacuole, cytoplasm, Golgi body and chloroplast. Protein sequence alignment of SsHAKs with their orthologs in sorghum showed that *S. spontaneum* and *Sorghum bicolor* shared identities ranging from 81 to 98%, with an average of 92.5% (Table 1). Four hundred thirty-five pairwise protein sequence comparisons among these SsHAKs showed that SsHAK19a and SsHAK19b shared the highest identity (96%), while other gene pairs had protein sequence similarities ranging from 28 to 82% with an average of 46%, indicating that the *SsHAK*s are an ancient gene family with high sequence divergence (Additional file 2).

**Fig. 1:**
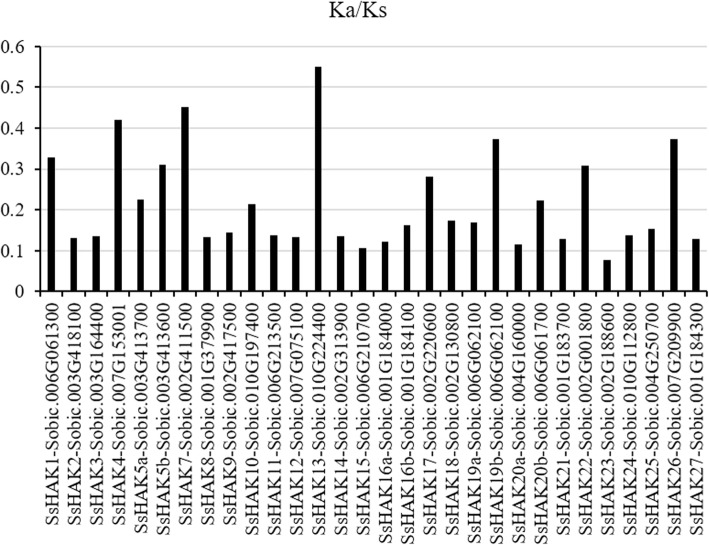
Nonsynonymous (Ka) and synonymous (Ks) substitution ratios of *SsHAKs* and their orthologs in sorghum. The Ka/Ks ratio was calculated by the Easy_KaKs calculation program (https://github.com/tangerzhang/FAFUcgb/tree/master/easy_Ka-Ks)

The nonsynonymous to synonymous substitution ratios (Ka/Ks) between *SsHAKs* and their orthologous genes in sorghum were calculated to study the evolutionary functional constraints in sugarcane. The results showed that the Ka/Ks ratios were less than 0.5, except for *SsHAK13*, suggesting that purifying selection was the main force driving the evolution of *HAK* genes (Fig. 1).

### Phylogenic analysis of *HAK* genes in *S. spontaneum* and representative angiosperms

**Fig. 2:**
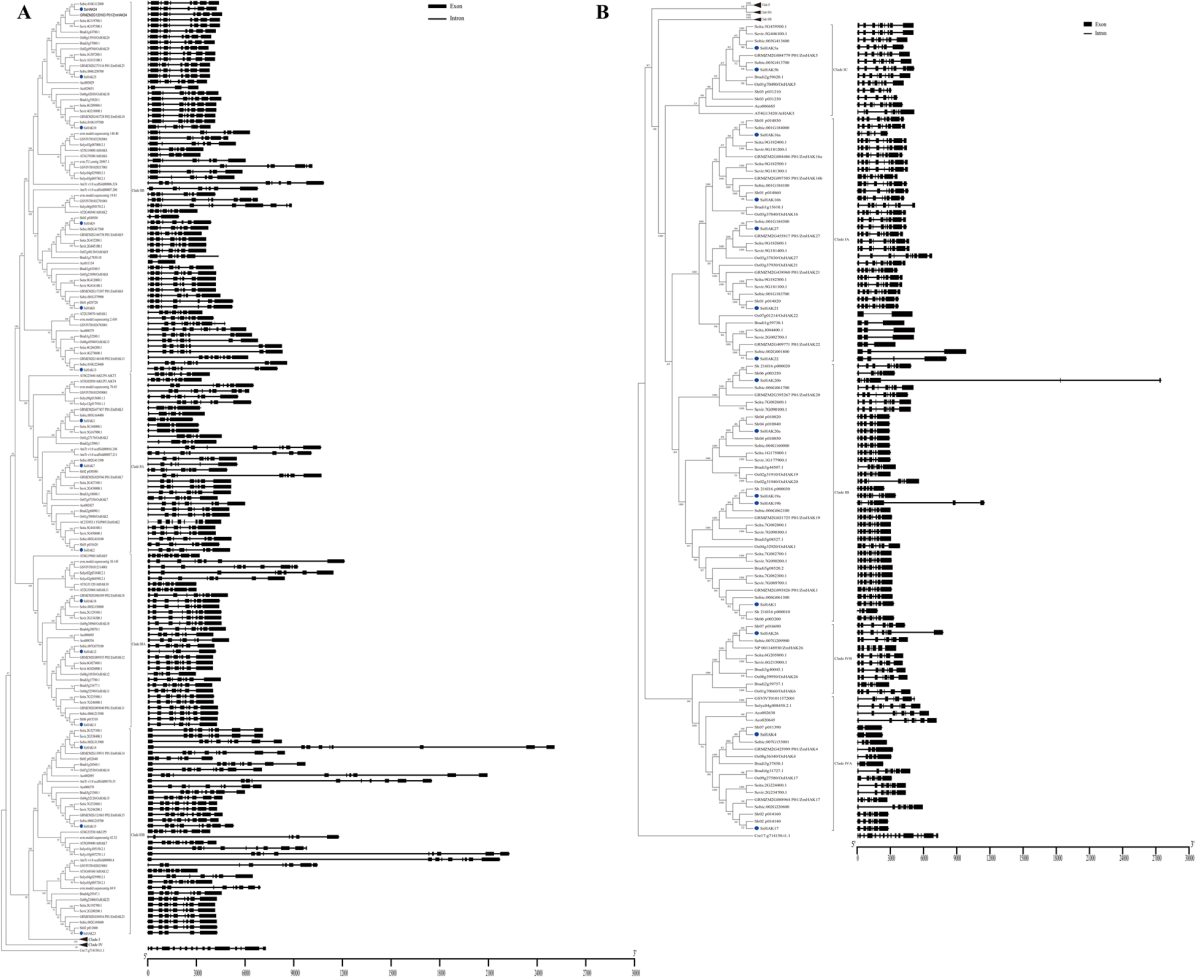
Phylogeny and schematic diagram for intron/exon organization of *HAK/KUP/KT* genes from 15 plant species. **a** Clade II and clade III. **b** Clade I and clade IV

To analyze the evolution of the *HAK* gene family in *S. spontaneum* and different plants, a total of 278 *HAK* genes from 14 representative angiosperms and a *HAK* member from *Chlamydomonas reinhardtii* as the outgroup were used to construct a phylogenetic tree using the neighbor-joining method (Fig. 2, Additional file 3). The 278 *HAK* genes included 6 from *Amborella trichopoda*, 8 from *Solanum lycopersicum*, 13 from *Vitis vinifera*, 8 from *Carica papaya*, 13 from *Arabidopsis thaliana*, 12 from *Ananas comosus*, 25 from *Brachypodium distachyon*, 27 from *Oryza sativa*, 28 from *Setaria italica*, 28 from *Setaria viridis*, 27 from *Zea mays*, 29 from *Sorghum bicolor*, 30 from *Saccharum spontaneum* and 24 from *Saccharum* hybrid R570 [29]. The amino acid sequence of the 279 HAK/KUP/KT transporters from 15 representative plant species is provided in the supplementary data (Additional file 4).

**Fig. 3:**
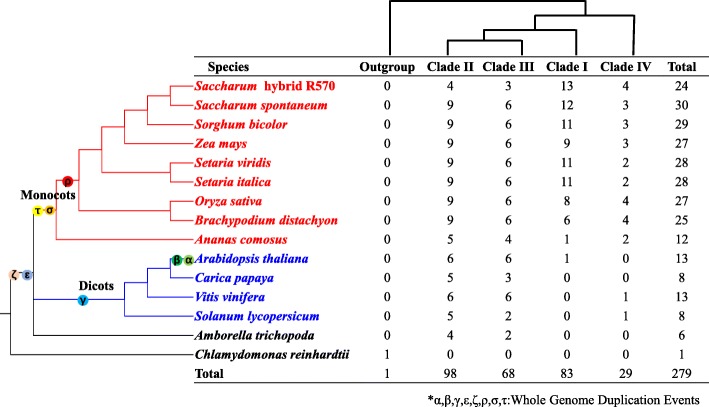
Phylogenetic relationships of *HAK/KUP/KT* families based on the current data for angiosperms

These *HAK* genes could be divided into four clades (I, II, III, IV) based on previously reported *OsHAKs* [17]. In *A. trichopoda*, the earliest diverging angiosperm, there were only 6 *HAK* genes, while in dicots and monocots, the number of *HAKs* ranged from 8 to 30 (Figs. 2 and 3), indicating that the ancient whole-genome duplication (WGD) contributed to the expansion of the *HAK* gene family in both dicots and monocots. Clade II and clade III included *HAK* genes from all 14 angiosperm genomes, indicating that the progenitors of these genes may have already existed prior to the split from angiosperms (Figs. 2 and 3). Clade I and clade IV mainly contained *HAK* genes from monocotyledons. Eighty-three *HAK* genes were identified in clade I, in which only one *HAK* gene was from *A. comosus* (Aco006685, homologous with *SsHAK5*) and *Arabidopsis* (*AtHAK5*), and the other 81 *HAK* genes were from all eight examined *Poaceae* species (Figs. 2 and 3). Twenty-nine *HAKs* were grouped into clade IV, and only 2 of them were from dicotyledons. These results indicated that the *HAKs* were unevenly distributed.

According to the Ks value in sorghum and sugarcane (Additional file 5), the divergence time of four clusters of *HAKs* was estimated. The median value of Ks was between 1.644 and 2.851, and its corresponding divergence time was between 134.8 and 233.7 Mya, indicating that the *HAK* was an ancient and divergent family. Furthermore, two pairs of duplicated *SsHAKs* (*SsHAK5a/5b* and *SsHAK16a/16b*) diverged at 18.94 and 58.14 Mya (Additional file 6). These results suggested that the *SsHAK* family is an ancient gene family with recent gene duplication events.

### Exon/intron organization of the *HAK* family in *S. spontaneum* and other angiosperms

To investigate the structural characteristics and evolution of the *HAK* gene family, the exon/intron organization in *HAKs* was mapped to the phylogenic tree, and the gene features and patterns were analyzed (Fig. 2). The exon number in the *HAK* family of the 15 examined plant species ranged from 2 to 16, with an average of 8.4, and 217 out of 279 (77.8%) *HAK* genes possessed 8 to 10 exons (Additional files 7 and 8). This result suggested that the last common ancestor (LCA) of angiosperm *HAK* genes had 8 to 10 exons.

The exon number of *SsHAKs* varied from 2 to 12, and half of the *SsHAKs* possessed 8 or 9 exons. The pattern of *SsHAK* gene structure was similar to that of *HAK* gene structure from sorghum and maize in the same clade, suggesting that the *HAK* gene structure in the *Panicoideae* was relatively conserved. In clade I, the exon number in *HAK* genes varied from 2 to 12, which was the most variation among these 4 clades. Notably, the *HAK* genes in the subfamily with *SsHAK22* had only 2 to 4 exons; however, the protein size remained consistent, which was likely due to the loss of introns. Clade II had the largest number of *HAK* genes, with 60 out of 98 *HAKs* possessing 9 exons and 5 out of 9 *SsHAKs* harboring 8 exons. *SsHAK3/8/10* had one less exon than their orthologous genes in sorghum; the first exon in *SsHAK13* and seventh exon in *SsHAK24* were smaller than the corresponding exons in sorghum, and both resulted in shorter amino acid sequences in *S. spontaneum* (Table 1, Fig. 2). In clade III, the exon number was relatively conserved, with 61 out of 68 *HAK* genes possessing 8 to 10 exons, while the gene size varied greatly, mainly due to the different sizes of introns. The exon number in clade IV ranged from 2 to 8, with an average of 7, which was smaller than that in other clades. Notably, the *HAK* genes in the subfamily with *SsHAK4* had only 2 to 5 exons, which was likely caused by intron loss during the process of evolution. The results indicated that *HAKs* underwent gene structure reconstruction under different evolutionary dynamics in *S. spontaneum* and other angiosperms in this study.

### Expression analysis of *HAK* genes in *Saccharum* species

To study the expression profiles and potential functions of *HAKs* in *Saccharum*, we compared the gene expression patterns according to 4 sets of RNA-seq data: 1) different developmental stages and tissues; 2) a leaf gradient; 3) the circadian rhythm; and 4) treatment under low-potassium stress. The FPKM values of *HAK1*, *HAK7* and *HAK20b* in YT55 at 0 h, 6 h, 12 h, 24 h, 48 h and 72 h under K^+^-starvation conditions were verified by RT-qPCR. The relative expression level was positively correlated with the FPKM value (*R*^2^ = 0.8419, Additional file 9), suggesting the reliability of gene expression based on the RNA-seq analysis.

### Expression pattern of *HAKs* in different tissues at different stages

**Fig. 4:**
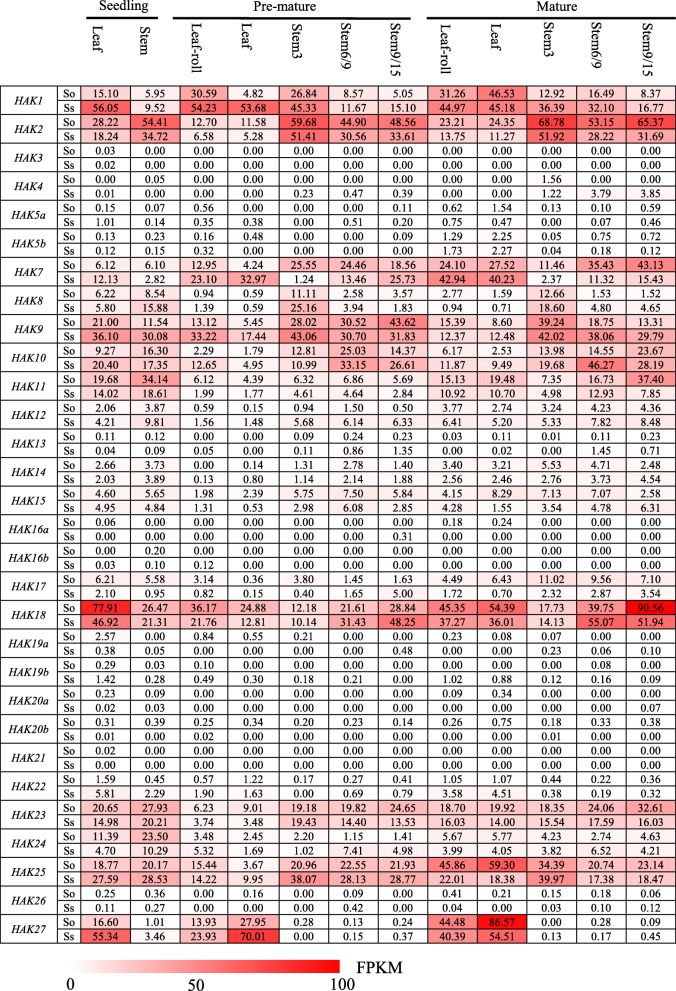
The expression pattern of *HAK/KUP/KT* genes based on FPKM in different tissues in different stages in *S. officinarum* and *S. spontaneum*

To study gene functional divergence among the *Saccharum* species, transcriptome profiles of *HAKs* between two *Saccharum* species, *S. officinarum* and *S. spontaneum,* were analyzed based on RNA-seq at three developmental stages (seedling, premature and mature stages) in five different tissues, 2 leaf (leaf and leaf roll) and 3 stalk (immature, maturing and mature) tissues (Fig. 4). Among the 30 *HAK* genes analyzed, 18 genes (*HAK3/4/5a/5b/12/13/14/15/16a/16b/17/19a/19b/20a/20b/21/22/26*) showed very low or undetectable expression levels in all examined tissues of the two *Saccharum* species. *HAK1* and *HAK2* had different expression patterns in the two *Saccharum* species. *HAK1* had higher expression levels in *S. spontaneum* than in *S. officinarum,* and the expression level in leaves was higher than that in stems at three different stages. *HAK2* had higher expression levels in *S. officinarum* than in *S. spontaneum*, and the expression level in stems was higher than that in leaves. *HAK8* was mainly expressed in the upper stems, while the expression levels in the middle and lower stems were very low. *HAK9* and *HAK10* had higher expression levels in stems than in leaves. *HAK18* was expressed in all examined tissues, with higher expression levels, especially in leaves at the seedling stage and in mature stems. Notably, *HAK27* was highly expressed in leaves at all examined three stages, but the expression level in stems was very low or undetectable.

### Expression pattern of *HAKs* across a leaf segment gradient

**Fig. 5:**
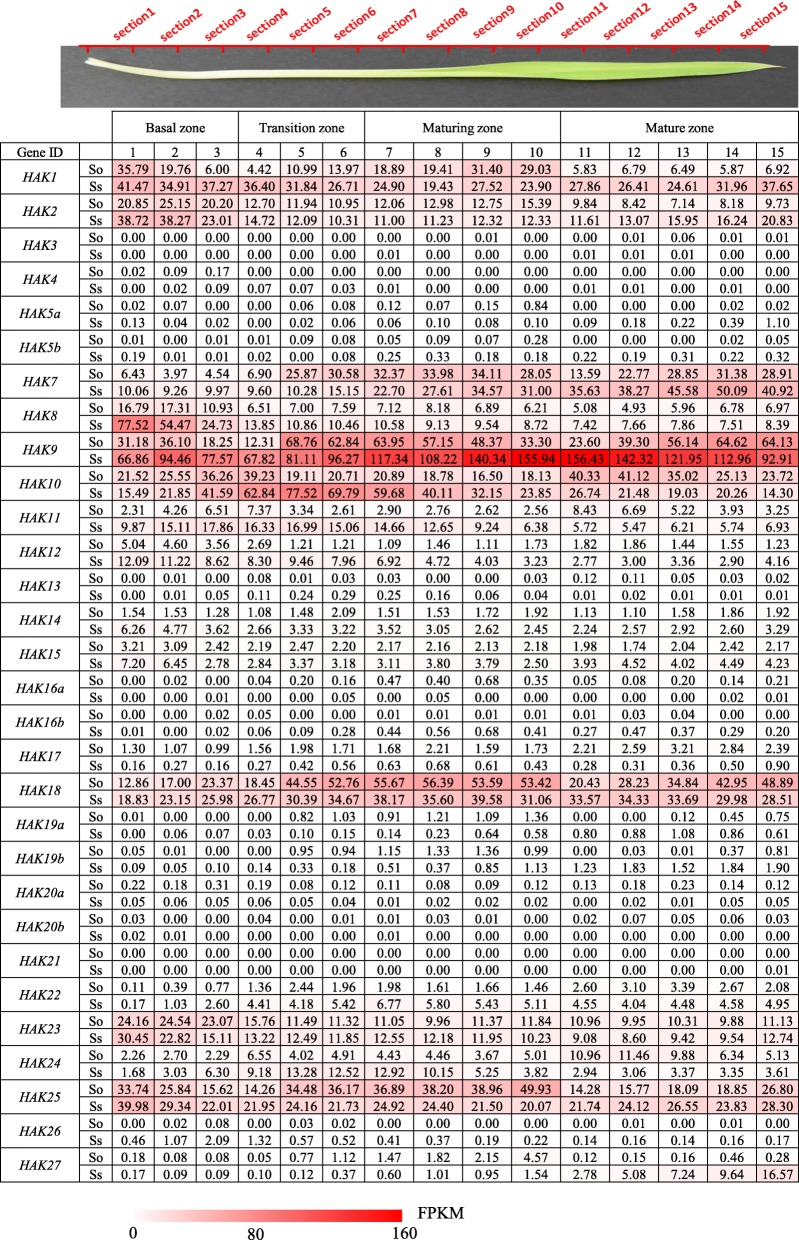
The expression pattern of *HAK/KUP/KT* genes based on FPKM across leaf gradients in *S. officinarum* and *S. spontaneum*

To further explore the functional divergence of *HAK* genes for photosynthesis in the source tissues, we studied the expression pattern of *HAKs* in continuously developing leaf segment gradients from *S. officinarum* and *S. spontaneum* (Fig. 5). *Saccharum* leaves were divided into four zones: the basal zone (sink tissue), transitional zone (undergoing sink-source transition), maturing zone and mature zone (fully differentiated zone with active photosynthesis), following the method described in maize [30]. Consistent with the expression pattern at different developmental stages, 18 *HAK* genes (*HAK3/4/5a/5b/12/13/14/15/16a/16b/17/19a/19b/20a/20b/21/22/26*) showed very low or undetected expression levels in all examined leaf segments, suggesting their limited roles in sugar transport (Fig. 5). *HAK1* and *HAK2* showed higher expression levels in the basal zone than in the other 3 zones. The expression level of *HAK7* increased gradually from the base to the tip of the *S. spontaneum* leaf, while in *S. officinarum*, *HAK7* displayed higher expression levels in the maturing zone than in the other 3 zones. The expression level of *HAK8* decreased gradually from the base to the tip of the leaf in both *S. officinarum* and *S. spontaneum*. *HAK9* showed different expression patterns in *S. spontaneum* and *S. officinarum*. In *S. spontaneum*, the expression level of *HAK9* increased gradually from the basal zone to the maturing zone and then decreased in the mature zone. In *S. spontaneum*, the expression level of *HAK9* decreased from the transition zone to the maturing zone and then increased in the mature zone, and the expression level was much higher in *S. officinarum*, suggesting gene functional divergence after the split of these two *Saccharum* species. *HAK10* showed higher expression levels in the transition zone in *S. spontaneum* and higher expression levels in the mature zone in *S. officinarum*. *HAK18* displayed higher expression levels in the maturing zone in both *S. spontaneum* and *S. officinarum*, while *HAK23* showed higher expression levels in the basal zone in the two *Saccharum* species. *HAK25* displayed higher expression levels in the maturing zone in *S. officinarum* but higher expression levels in the basal zone in *S. spontaneum*.

### Expression pattern of *HAKs* during the circadian rhythm

**Fig. 6:**
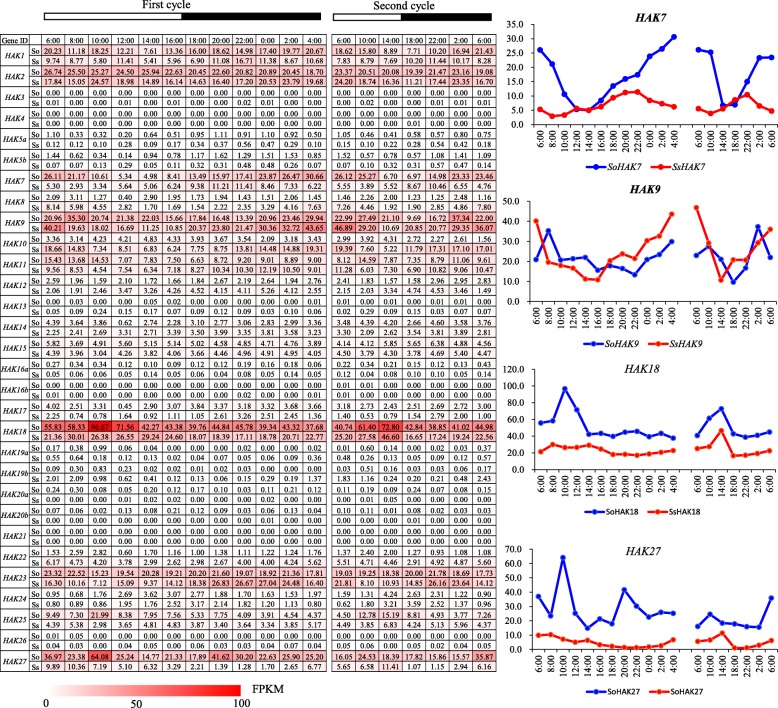
The expression pattern of *HAK/KUP/KT* genes based on FPKM during the diurnal cycles in *S. officinarum* and *S. spontaneum*

Acting as an enzyme activator, potassium ions participate in a series of photosynthetic processes [31]. To analyze the expression pattern of *HAKs* during diurnal cycles, we investigated the transcriptome profiles of the mature leaves in the two *Saccharum* species at 2 h intervals over a 24 h period and at 4 h intervals over an additional 24 h. Consistent with the transcriptome profiles at different developmental stages and in the leaf segment gradient, 18 genes (*HAK3/4/5a/5b/12/13/14/15/16a/16b/17/19a/19b/20a/20b/21/22/26*) displayed very low or undetectable expression levels in the two *Saccharum* species, further supporting their limited roles in growth and development (Fig. 6). In addition, *HAK8* and *HAK24* also showed low expression levels over the two 24 h periods. *HAK1*, *HAK2*, *HAK7*, *HAK18* and *HAK27* showed higher expression levels in *S. officinarum* than in *S. spontaneum*, while *HAK9* and *HAK10* displayed higher expression levels in *S. spontaneum* than in *S. officinarum*. *HAK1* and *HAK2* had no diurnal expression pattern in the two *Saccharum* species. *HAK7* displayed a higher expression level at night than in the daytime and showed the lowest expression level at noon in *S. officinarum* but showed no diurnal expression pattern in *S. spontaneum*. *HAK10* displayed a higher expression level at night than in the daytime in *S. spontaneum* but showed no diurnal expression pattern in *S. officinarum. HAK9* displayed a higher expression level at night than in the daytime in both *Saccharum* species*. HAK18* and *HAK27* displayed higher expression in the morning in the two *Saccharum* species. These findings suggested the functional divergence of the *HAK* genes in diurnal rhythms.

### Expression pattern of *HAKs* under K^+^-deficiency stress

**Fig. 7:**
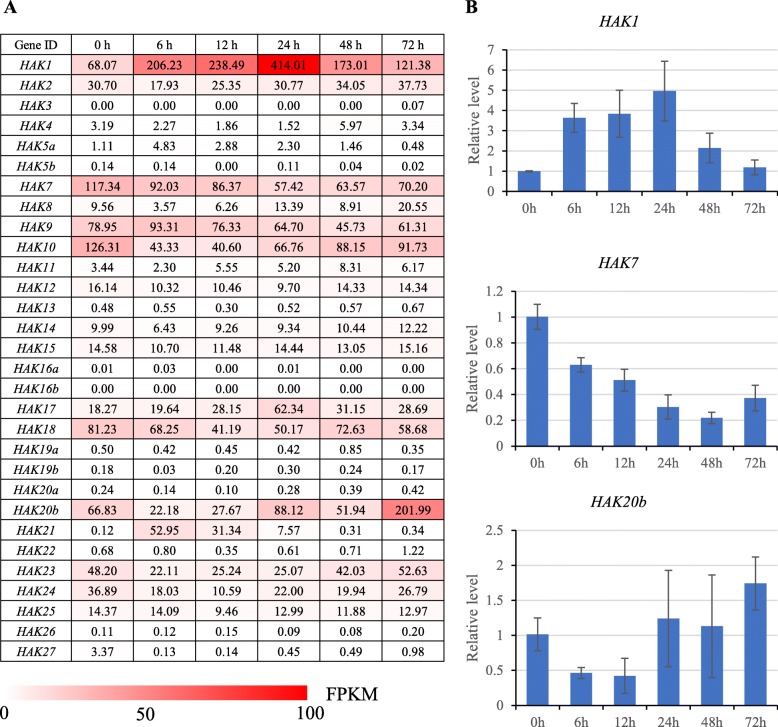
**a** The expression pattern of *HAK/KUP/KT* genes in *Saccharum* hybrid YT55 under low-K^+^ stress conditions based on FPKM values. **b** The relative expression level detected by RT-qPCR

To investigate the functional divergence of *HAK* genes in response to low-potassium stress in sugarcane, we studied the expression profiles of *HAKs* in roots from the *Saccharum* hybrid variety YT55 at 0 h, 6 h, 12 h, 24 h, 48 h and 72 h under low-K^+^ stress (Fig. 7). Among the 30 *HAK* genes analyzed, 14 genes (*HAK3/4/5a/5b/11/13/16a/16b/19a/19b/20a/22/26/27*) showed very low or undetectable expression levels before and after exposure to low-K^+^ stress. Notably, *HAK1* showed strong induction in roots under low-K^+^ conditions, reached the highest level at 24 h, and then decreased subsequently at 48 h and 72 h. *HAK21* was strongly induced after exposure to low-K^+^ stress within 12 h but was subsequently downregulated to a low expression level. *HAK20b* was downregulated within 12 h and then upregulated to the highest level at 72 h. *HAK7*, *HAK10*, *HAK18* and *HAK24* were downregulated after exposure to low-K^+^ stress. Other *HAKs*, such as *HAK12/14/15/25*, were constitutively expressed.

### Functional complementation validation of *SsHAK1* and *SsHAK21* in the yeast mutant strain R5421

**Fig. 8:**
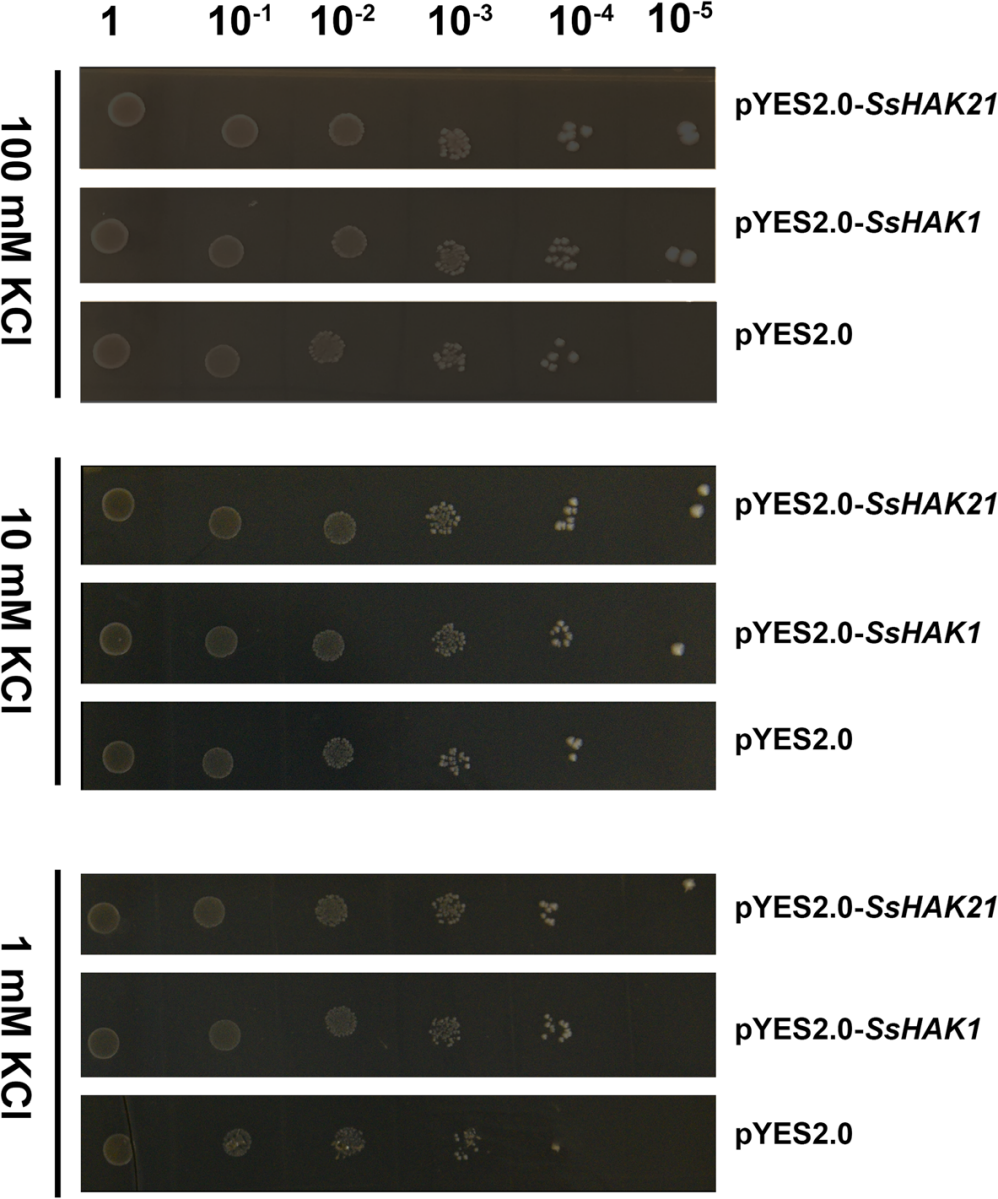
Phenotypic identification of the yeast mutant strain R5421 transformed with *SsHAK1* or *SsHAK21*

*SsHAK1* and *SsHAK21* were selected for complementary validation in yeast because they were both induced in response to low-K^+^ stress. The transformed yeast strain carrying only the empty vector pYES2.0 was used as a control. There were no obvious growth differences between yeast transformed with pYES2.0 and yeast transformed with pYES2.0-*SsHAK1* or pYES2.0-*SsHAK21* in SC/−ura medium containing 100 mM KCl (Fig. 8). However, when the KCl concentration decreased to 10 mM, the growth of yeast transformed with *SsHAK1* and *SsHAK21* was better than that of yeast transformed with the empty vector. When the KCl concentration decreased to 1 mM, the growth of yeast transformed with the empty vector was significantly inhibited, while the growth of yeast transformed with *SsHAK1* or *SsHAK21* was almost unaffected (Fig. 8). These results suggested that both *SsHAK1* and *SsHAK21* could recover the K^+^ absorption function in the yeast mutant strain R5421, indicating that they had potassium transporter activity.

## Discussion

The *HAK/KUP/KT* family of potassium transporters have been widely reported to be associated with K^+^ transport across membranes in plants. They also play vital roles in response to salt and drought stress, as well as morphogenesis of root and shoot [7]. However, genome-wide analysis of the *HAK/KUP/KT* gene family has not been conducted in *Saccharum* due to its complex genetic background. The recently released *S. spontaneum* genome allowed us to identify 30 *HAK* genes from *S. spontaneum*. In addition, 248 *HAK* genes from 13 other representative plant species and an outgroup were used to construct a phylogenetic tree and study the evolution of *HAK* genes in *Saccharum*. Furthermore, expression analysis based on RNA-seq revealed spatiotemporal expression and functional divergence in the *HAK* family, which provides valuable information and robust candidate genes for future functional analysis.

### Evolution of the *HAK* gene family in *Saccharum* and representative angiosperms

WGD or polyploidization, gene loss and diploidization are considered important evolutionary forces in plants [32, 33]. Angiosperms, pancore eudicots and monocots originated from ε, γ and σ WGD events, which have been revealed by a rigorous phylogenomic approach [33]. A recent study showed that pineapple had one fewer ancient ρ WGD events than other gramineous plants [34]. *A. trichopoda* is the earliest known angiosperm to have evolved separately from other angiosperms and has attracted much attention from botanists. In this study, 279 *HAKs* from 15 plant species representing major WGD events in angiosperms, together with the WGD information, allowed us to study *HAK* gene evolution. *HAKs* from different plant species could be divided into four clades in in descending order of duplications: clade IV, clade I, clade III and clade II. Based on the estimated divergence time among the 4 clades of the *SsHAK* gene family (134.8 to 233.7 Mya, Additional file 5), the *SsHAK* family in angiosperms probably occurred before the σ WGD event in angiosperms (approximately 130 Mya) and after the ε WGD event (approximately 220 Mya) [33].

The number of *HAKs* in the four clades varied greatly (from 29 to 98, Fig. 3), which is consistent with a previous study in which *HAKs* were unevenly distributed in different clades among angiosperms [35]. In clade I, only one *HAK* gene member was from *A. comosus* and *Arabidopsis*, while in *Poaceae* species, the *HAK* number ranged from 6 to 13. This result indicated that WGD or recent gene duplication contributed greatly to the expansion of *HAKs*. *SsHAK5a/5b*, *SsHAK16a/16b*, and *SsHAK19a/19b* were from tandem duplications, while *SsHAK20a/20b* may have originated from a transposed duplication. The LCAs of *SsHAK5* and *SsHAK18* (in clade III) may have occurred before the split of monocotyledonous and dicotyledonous plants. *HAK5* was speculated to be lost in other dicotyledons except for Arabidopsis, which may be due to the gene functional redundancy of the *HAK* family. *HAK18* was retained in all monocotyledonous and dicotyledonous plants, showing its functional constraint for the *HAK* family, and the expression profile analysis of *HAK18* also confirmed this.

In clade II and clade III, *SsHAK2* and *SsHAK7* were retained from the ε WGD event, and in dicotyledons, these two orthologous genes were lost. *SsHAK3* and *SsHAK13* originated after *A. trichopoda* had evolved separately from other angiosperms. *SsHAK8*, *SsHAK9* and *SsHAK10* were assumed to be retained from the ε WGD event; *SsHAK11*, *SsHAK12*, *SsHAK15*, *SsHAK24* and *SsHAK25* were retained from the σ WGD event, as only monocotyledons contained these genes. *SsHAK14* and *SsHAK23* were assumed to be retained from the ε WGD event, but *HAK14* was probably lost in dicotyledons. Clade IV contained the lowest number of *HAKs*. *SsHAK4* and *SsHAK17* originated before the split of monocotyledons and dicotyledons and after the split of *A. trichopoda* from angiosperms. The LCA of *SsHAK26* originated after the split of the *Gramineae* and pineapple.

The *HAK* gene family in plants exhibited a less conserved exon/intron structure. The exon number in *Saccharum* ranged from 2 to 12 (Fig. 1, Additional file 7), and the variation range in *Saccharum* was larger than that in rice [17], maize [19] and wheat [36]. Three types of mechanisms, exon/intron gain/loss, exonization/pseudoexonization and insertion/deletion, mainly led to exon-intron structure differences in paralogous or orthologous genes [37]. Although the gene structure of *SsHAKs* changed greatly, the protein size was relatively conserved, suggesting that exon-intron structure differences in *SsHAKs* were mainly caused by intron gain/loss. Clade I and clade IV belong to the older *HAK* family in *Saccharum*, so the *HAKs* in these two clades were speculated to have more intron gain/loss events based on the ‘introns-early’ theory during the lengthy evolutionary process [38, 39]. The results in this study also support this view because the variation in exon number in clade I and clade IV was much greater than that in clade II and clade III.

### Gene expression and functional divergence of *HAKs* in *Saccharum*

The transcriptional regulation of K^+^ transporters is a common mechanism by which plant species respond to low-K^+^ stress [8], and expression pattern analysis can provide insight into the potential functions of the *HAK* gene family. In this study, we found that most *HAK* genes in clade I and clade IV showed low or undetectable expression levels across all examined samples. Most *HAK* genes in clade II and clade III were strongly expressed in all tested tissues. These results were consistent with the results of previous studies on *HAK* genes in rice [17], Arabidopsis [25] and wheat [36]. Five *OsHAK* genes (*OsHAK2/10/15/23/25*) from clades II and III were expressed in all examined tissues of three different genotypes [17]. In Arabidopsis, 12 out of 13 *HAK/KUP/KT* genes were from clades II and III, most of which were expressed in the roots, leaves, siliques and flowers [25]. Similarly, most *TaHAKs* in wheat belonging to clades II and III were constitutively expressed in all tissues [36].

Low-K^+^ stress tends to induce the upregulated expression of K^+^ transporter genes [40]. Previous studies demonstrated that the expression of *OsHAK1* in rice [20], *TaHAK1* in wheat [36] and *PbrHAK1* in pear [41] was induced by K^+^ starvation. In this study, the expression level of *SsHAK1* increased rapidly under low-K^+^ stress, and this result is in good agreement with previous studies. Notably, *SsHAK21* was substantially upregulated after a short period of K^+^-starvation treatment and then rapidly downregulated (transient activation), indicating that *SsHAK21* was involved in the low-K^+^ stress response in sugarcane. Similar results were found in rice, as *OsHAK21* functions in the maintenance of ion homeostasis and tolerance to salt stress [42]. *SsHAK1*, *SsHAK17* and *SsHAK21* displayed upregulated expression, suggesting that they may play important roles in maintaining normal growth and mediating potassium acquisition under K^+^ deficiency. In addition, nearly half of the *SsHAK* genes were not expressed or had very low levels of expression in all tested tissues at all stages or even under low-K^+^ stress, which may be caused by the gene functional redundancy due to WGD events in sugarcane.

**Fig. 9:**
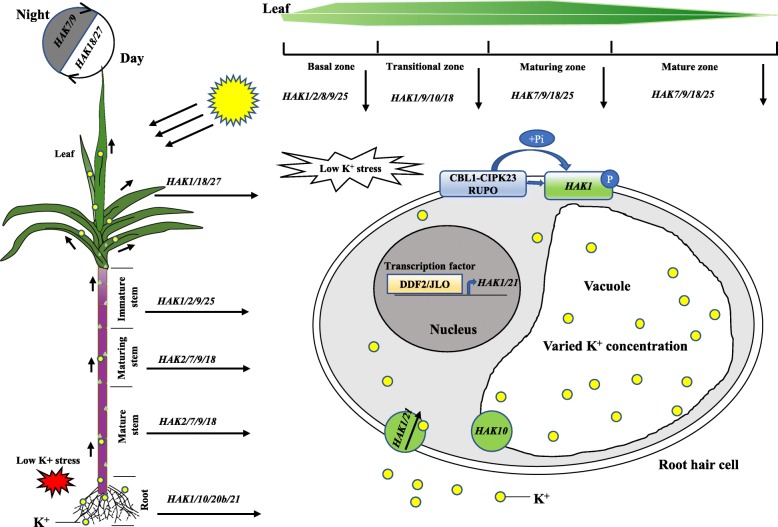
Schematic models for the roles of *HAKs* based on gene expression profiles in sugarcane. In the maturing and mature zones of the leaves and stems, *HAK7/9/18* were the main expressed genes. Moreover, these genes also presented a diurnal expression pattern. *HAK25* was mainly expressed in the maturing and mature zone of leaf tissues, while *HAK2* was mainly expressed in the stem. Low-K^+^ stress induced the upregulation of the expression of *HAK1* and *HAK21*. Transcription factors such as DDF2 and JLO may directly bind to the promoters of *HAK1/21* to induce gene expression and subsequently promote HAK transporters, such as HAK1 and HAK 21, to acquire K^+^ in roots. HAK1 may be phosphorylated and activated by the CBL1-CIPK23 complex or receptor-like kinase, RUPO (ruptured pollen tube). The K^+^ concentration in the vacuole is highly varied to maintain cellular K^+^ homeostasis. Some HAK transporters, such as HAK10, located in the tonoplast of vacuoles may play a role in regulating the K^+^ concentration in vacuoles

The root system acquires K^+^ from the soil solution, and then K^+^ is transported among compartments within cells and from the roots to the shoots. A schematic model was proposed based on the expression profiles of the 30 *SsHAK* genes to illustrate the spatial and temporal gene expression in plant tissues and root hair cells of sugarcane (Fig. 9). *HAK7/9/18* were mainly expressed in the tissues of maturing and mature stems and leaves, indicating their important roles in K^+^ transport in these tissues. *HAK7/9/18/25* also showed a circadian rhythm expression pattern, suggesting that these genes were regulated by sunlight. Low-K^+^ stress induced the upregulation of the transcriptional expression of *HAK* genes. In Arabidopsis, transcription factors, such as DDF2, JLO, ARF2, RAP2.11, TFII_A, and bHLH121, directly bind the promoter of *AtHAK5* to induce its expression and increase tolerance to low-K^+^ and salt stress [26]. In this study, the expression of *HAK1* and *HAK21* was greatly upregulated, which may also be positively regulated by transcription factors (TFs), such as DDF2 and JLO, and further experiments, such as yeast one-hybrid assays, can be used to screen the TFs. AtHAK5 and its homologs from pepper and tomato can be activated by the CIPK23 (CBL-interacting protein kinase 23)/CBL1 (calcineurin B-like protein) complex [27]. In rice, OsHAK1/19/20 can be phosphorylated by a receptor-like protein kinase, RUPO (ruptured pollen tube) [43]. In this study, the CBL-CIPK complex and the receptor-like kinase RUPO may also act as regulators of high-affinity potassium transporters, such as HAK1, via phosphorylation-dependent interactions.

## Conclusions

In this study, 30 *HAK* (high-affinity K^+^ transporter) genes were identified through comparative genomics analyses of sugarcane. Evolutionary analysis revealed that both ancient whole-genome duplication (WGD) and recent gene duplication contributed to the expansion of the gene family, and purifying selection was the main force driving evolution. *HAK/KUP/KT* genes were accompanied by intron gain/loss in the process of evolution. Expression analysis based on RNA-seq under low-K^+^ stress and at different developmental stages revealed spatiotemporal expression and functional divergence in the *HAK/KUP/KT* gene family. Yeast functional complementation analysis showed that *SsHAK1* and *SsHAK21* mediated K^+^ transport under low-K^+^ stress. Altogether, these results provide valuable information and robust candidate genes for future functional analyses for the genetic improvement of potassium-utilization efficiency in sugarcane.

## Methods

### Plant materials

Two *Saccharum* species, LA-Purple (*S. officinarum*, 2n = 8x = 80, originated in the USA and was introduced into China; the plants were provided by Zhang’s laboratory at Fujian Agriculture and Forestry University) and SES-208 (*S. spontaneum*, 2n = 8x = 64, originated in the USA and was introduced into China; the plants were provided by Zhang’s laboratory in Fujian Agriculture and Forestry University), were cultivated at Fujian Agricultural and Forestry University (Fuzhou, 119°16′48″E, 26°4′48″N, Fujian, China) and sampled for gene expression pattern analysis.

The K^+^ uptake-deficient yeast mutant strain R5421 (*ura3–52 his3Δ200 leu2Δ1 trp1Δ1 ade2 trk1Δ::HIS3 trk2Δ::HIS3*) was provided by Professor Guohua Xu from Nanjing Agricultural University. R5421 cannot grow normally when the external potassium concentration is below 10 mM. *E. coli DH5α* competent cells and the expression vector pYES2.0 were purchased from TaKaRa Biotechnology Co., Ltd. (Dalian, China).

For expression pattern analysis at different developmental stages, tissue samples including leaf roll, leaf (fully expanded leaf), top immature internode, premature internode and mature internode were collected from premature plants (9-month-old plants) and mature plants (12-month-old plants). The sugarcane internodes were numbered from top to bottom. Leaf and stem tissues in the seedling stage were collected from 35-day-old plants as previously described [44, 45].

For expression pattern analysis of the leaf gradient, the two *Saccharum* species were grown in a greenhouse with light intensities of 350 μmol/m^2^/sec, 14:10 L/D, 30 °C L/22 °C D and 60% relative humidity. The second leaf of 15-day-old LA-Purple and 11-day-old SES208 after planting at 3 h into the light period and samples preparation method was as described by Li et al. [30].

For expression pattern analysis of the diurnal cycle, leaves of the mature plants of LA-Purple and SES208 were sampled consecutively 12 times with 2 h intervals from 6:00 a.m. on March 2, 2017, then sampled consecutively 7 times with 4 h intervals from 6:00 a.m. on March 3, 2017. The sunrise and sunset times on March 2, 2017 in Fuzhou were 6:25 a.m. and 6:05 p.m. respectively. Tissue collection was performed following a previously described method [34].

For expression pattern analysis under low-potassium stress, *Saccharum* hybrid variety YT55 (this variety was bred by Guangzhou Sugarcane Industry Research Institute and was planted in breeding bases for sugarcane in Wengyuan, Guangdong Province) was cultured at a normal potassium level (3.0 mmol /L) for 20 days in a greenhouse and then transferred to the K^+^-deficient nutrient solution (0.1 mmol /L) for starvation treatment. Mixed samples of roots from 6 plants in a pot (a biological replicate and three biological replicates in total were collected) were collected at 0 h, 6 h, 12 h, 24 h, 48 h and 72 h after starvation and stored in liquid nitrogen for total RNA isolation.

### Homology search analysis

According to previous reports, the protein sequences of 13, 27 and 27 *HAK/KUP/ KT* gene families identified in *Arabidopsis thaliana*, *Oryza sativa* and *Zea mays* [17–19] were obtained from Phytozome V12.1 (https://phytozome.jgi.doe.gov/pz/portal.html). With these protein sequences as queries, putative members of the *HAK/KUP/ KT* gene family were searched using the BLASTP program in 14 representative angiosperm genomes, 9 monocotyledons (*Saccharum* hybrid R570 [29], *Saccharum spontaneum*, *Sorghum bicolor*, *Zea mays*, *Setaria viridis*, *Setaria italica*, *Oryza sativa*, *Brachypodium distachyon* and *Ananas comosus*), 4 dicotyledons (*Arabidopsis thaliana*, *Carica papaya*, *Vitis vinifera*, and *Solanum lycopersicum*) and *Amborella trichopoda*. Sequences with an e-value <1e^− 10^ were selected as HAK/KUP/KT candidates. Then, the identified HAK/KUP/KT proteins were subjected to conserved domain validation with the PFAM (https://pfam.xfam.org) and CDD (https://www.ncbi.nlm.nih.gov/Structure/bwrpsb/bwrpsb.cgi) databases. In addition, a *HAK* gene from *Chlamydomonas reinhardtii* was selected as the outgroup.

### Sequence and phylogenetic analyses

Isoelectric points (pI) and relative molecular weight of the HAK/KUP/KT proteins were predicted by ExPASy (https://web.expasy.org/compute_pi/). The exon-intron structures were assessed with TBtools [46]. TMHHM Server v.2.0 (http://www.cbs.dtu.dk/ services/TMHMM/) was used to predict the transmembrane domains of the HAK/KUP/KT proteins. Subcellular locations of the HAK/KUP/KT proteins were predicted by WoLF PSORT (https://www.genscript.com/wolf-psort.html).

The evolutionary history of 14 representative angiosperms was inferred by the neighbor-jointing (NJ) method [47]. Based on the protein sequence alignment, the phylogenetic tree of the *HAK/KUP/KT* gene family was constructed using NJ methods. The construction of the NJ tree was performed using MEGA7 [48] with the “pair deletion” and “Poisson correction” models. The reliability of the internal branches of the tree was evaluated by a bootstrap test (1000 replicates), and the percentages are shown next to the branches.

The nonsynonymous substitution ratios (Ka), synonymous substitution ratios (Ks) and Ka/Ks ratios of the 30 pairs *HAK/KUP/KT* orthologous genes from sorghum and sugarcane were calculated by the Easy_KaKs calculation program (https://github.com/tangerzhang/FAFU-cgb/tree/master/easy_KaKs). Fisher’s exact test for small samples was applied to verify the validity of Ka and Ks calculated by this method [49]. The divergence time (T) was calculated as T = Ks/ (2 × 6.1 × 10^− 9^) × 10^− 6^ Mya [50].

### Analysis of the expression profiling of *HAKs* in *Saccharum* based on RNA-seq

RNA preparation, cDNA libraries construction and RNA-seq libraries sequencing were performed as previously described [51, 52]. Raw data were aligned to available *S. spontaneum* AP85–441 reference gene models using Trinity (https://github.com/trinityrnaseq/trinityrnaseq/wiki). Fragments per kilobase per million mapped fragments (FPKM) values were calculated to represent gene expression levels as previously described [51, 52].

### Validation of *HAK* gene expression levels by RT-qPCR

The expression level of three *HAK* genes (*HAK1*, *HAK7* and *HAK20b*) in the roots of *Saccharum* hybrid variety YT55 at 6 time points (0 h, 6 h, 12 h, 24 h, 48 h and 72 h) under K^+^-starvation conditions was validated by RT-qPCR, to normalize the expression levels, 2 constitutively expressed genes, the *eukaryotic elongation factor 1a* (*eEF-1a*) and *actin* were used as reference genes, each sample had 3 biological replicates and 3 technical replicates. (Additional file 10). The reaction program of reverse transcription, real-time PCR and the relative expression levels calculation were carried out as Wang et al. described [52].

### Yeast expression vector construction and function complementation experiment of *SsHAK1* and *SsHAK21*

Primer Premier 5 was used to design primers (Additional file 11), and the synthesized cDNA from RNA of YT55 after 12 h of low-potassium stress treatment was used as a template to amplify *SsHAK1* and *SsHAK21*. The amplified products were recovered from the gel and ligated to the expression vector pYES2.0 with In-Fusion enzyme (TaKaRa Biotechnology Co., Ltd., Dalian, China). The ligation products were transformed into *E. coli* competent *DH5α* cells. Positive monoclonal clones were selected and verified by sequencing, and then the plasmids were extracted for subsequent yeast transformation. Competent cells of yeast mutant strain R5421 were prepared with the S.c. EasyComp™ Transformation Kit (Invitrogen, Carlsbad, CA, USA) and transformed. Yeast strains with the empty vector and target genes were isolated and then used for gradient dilution and inoculated in SC/−ura medium with 100 mM, 5 mM and 0 mM KCl. The results were observed after 3–5 days of culture at 30 °C.

## Supplementary information

**Additional file 1. **The *HAK* gene alleles in *Saccharum spontaneum*.

**Additional file 2.** Similarity between HAK proteins in sugarcane calculated by NCBI BLASTP.

**Additional file 3. **Phylogenetic relationships among the *KT/HAK/KUP* gene families from 15 representative plant species.

**Additional file 4.** Amino acid sequence of 279 HAK/KUP/KT transporters from 15 representative plant species.

**Additional file 5. **Divergence time among the 4 clades of the *HAK* family in *Sorghum bicolor* and *Saccharum spontaneum*.

**Additional file 6. **Divergence between paralogous *SsHAK* gene pairs in *Saccharum spontaneum*.

**Additional file 7. **Statistics of exon number in each *HAK*.

**Additional file 8. **The proportion of different numbers of exons in all *HAKs* from 15 plant species.

**Additional file 9. **Correlation coefficient between RNA-seq data and RT-qPCR of *HAK1*, *HAK7* and *HAK20b*.

**Additional file 10. **The primers for the RT-qPCR verification of four *HAK* genes in *Saccharum* hybrid YT55.

**Additional file 11. **The primers used to clone *SsHAK1* and *SsHAK21* and construct the yeast expression vector.

## Data Availability

The datasets supporting the conclusions of this article are included in the article and its additional files.

## Abbreviations

bHLH121: Basic helix-loop-helix 121
CBL: Calcineurin B-like protein
CIPK: CBL-interacting protein kinase
DDF2: Dwarf and delayed flowering 2
FPKM: Fragments per kilobase per million mapped fragments
HAK/KUP/KT: High-affinity K^+^ transporter/K^+^ uptake permease/K^+^ transporter
JLO: Jagged lateral organs
Ka: Nonsynonymous substitution ratio
Ks: Synonymous substitution ratio
LCA: Last common ancestor
RT-qPCR: Reverse transcription-quantitative PCR
TF: Transcription factor
TFII_A: Transcription initiation factor II_A gamma chain
WGD: Whole-genome duplication
