# Supplementary figures and images for: Genome-wide systematic characterization of the HAK/KUP/KT gene family and its expression profile during plant growth and in response to low-K+ stress in Saccharum
Source: BMC Plant Biol. 2020 Jan 13;20:20. doi: 10.1186/s12870-019-2227-7 (PMC6958797; doi:10.1186/s12870-019-2227-7)

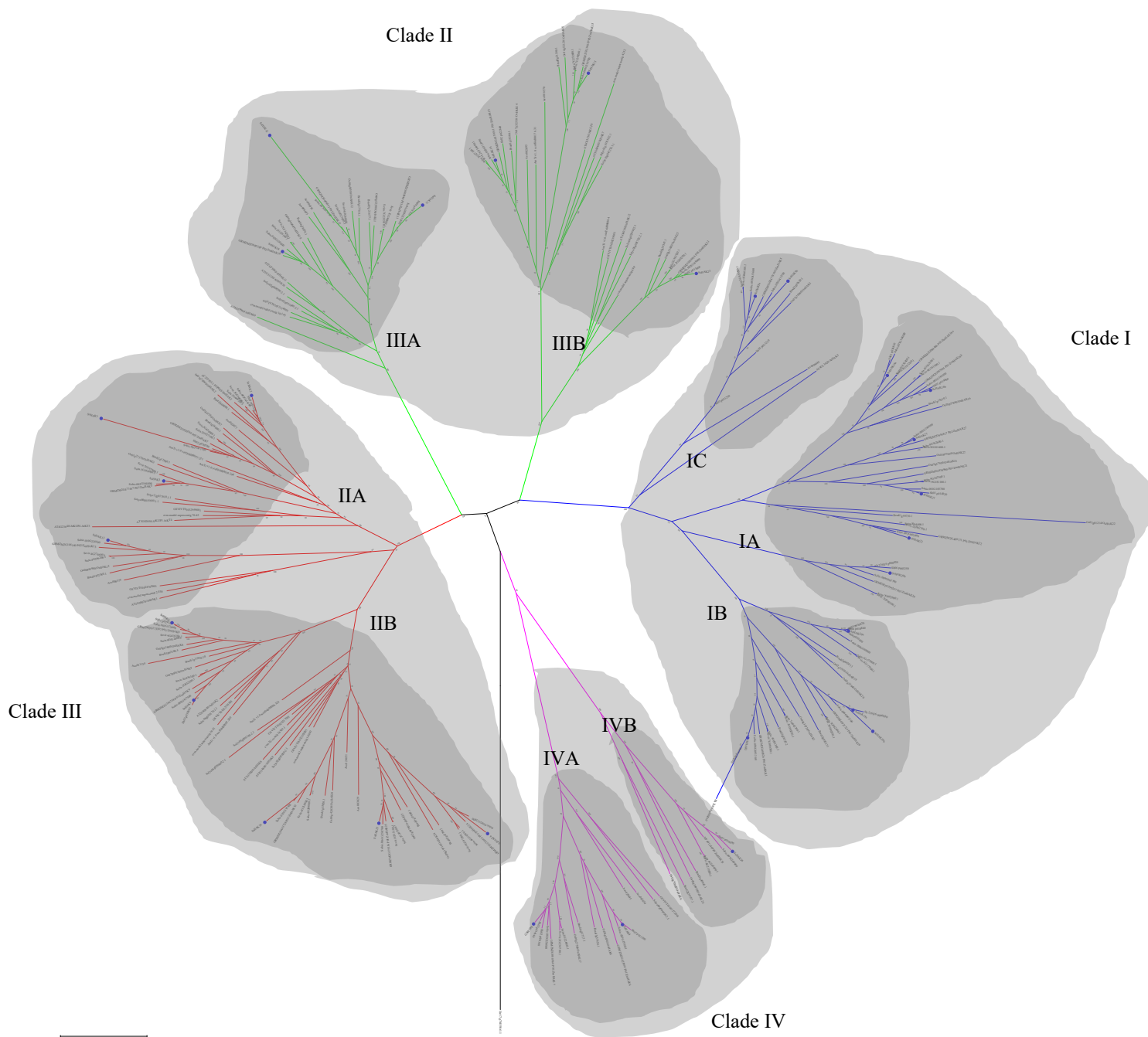

Supplement: Supplementary file 3 — Additional file 3. Phylogenetic relationships among the KT/HAK/KUP gene families from 15 representative plant species. [file 12870_2019_2227_MOESM3_ESM.pdf]

**Additional file 9:** Correlation coefficient between RNA-seq data and RT-qPCR of *HAK1*, *HAK7* and *HAK20b.*


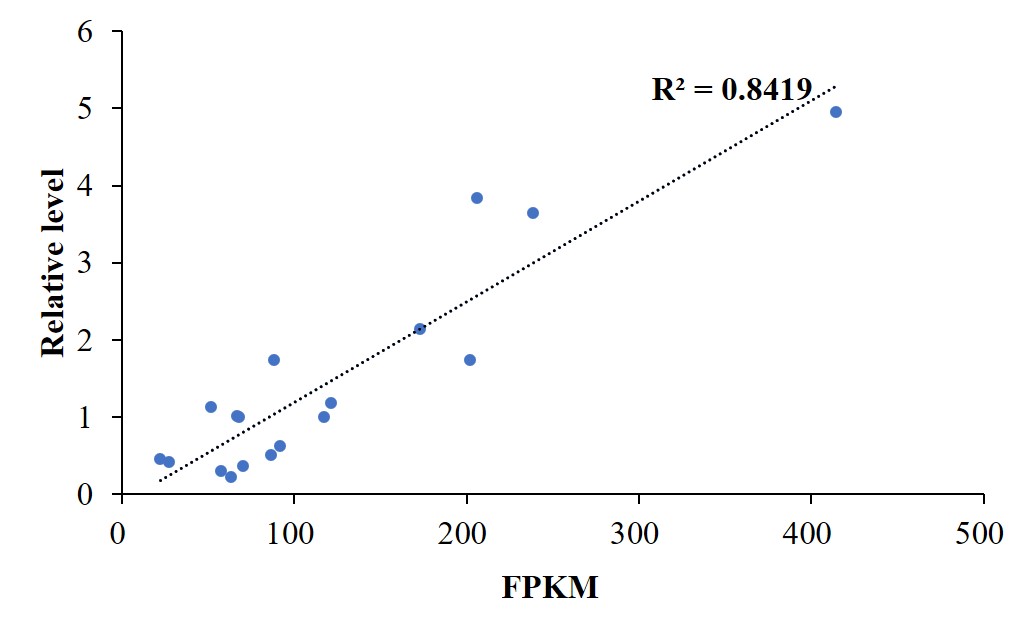

Supplement: Supplementary file 9 — Additional file 9. Correlation coefficient between RNA-seq data and RT-qPCR of HAK1, HAK7 and HAK20b. [file 12870_2019_2227_MOESM9_ESM.docx]
