# Supplementary material for: Genome-wide systematic characterization of the HAK/KUP/KT gene family and its expression profile during plant growth and in response to low-K+ stress in Saccharum
Source: BMC Plant Biol. 2020 Jan 13;20:20. doi: 10.1186/s12870-019-2227-7 (PMC6958797; doi:10.1186/s12870-019-2227-7)
Supplement: Supplementary file 5 — Additional file 5. Divergence time among the 4 clades of the HAK family in Sorghum bicolor and Saccharum spontaneum. [file 12870_2019_2227_MOESM5_ESM.docx]

**Additional file 5:** Divergence time among 4 clades of *HAK* family in *Sorghum bicolor* and *Sacchrum spontaneum*.

| Clade-Clade | Median Ks | Gene pairs used | Divergence time (Mya) |
| --- | --- | --- | --- |
| Clade I-Clade II | 1.804 | 414 | 147.9 |
| Clade I-Clade III | 2.851 | 276 | 233.7 |
| Clade I-Clade IV | 1.993 | 138 | 163.4 |
| Clade II-Clade III | 2.248 | 216 | 184.3 |
| Clade II-Clade IV | 1.644 | 108 | 134.8 |
| Clade III-Clade IV | 2.182 | 72 | 178.9 |
