# Supplementary material for: Genome-wide systematic characterization of the HAK/KUP/KT gene family and its expression profile during plant growth and in response to low-K+ stress in Saccharum
Source: BMC Plant Biol. 2020 Jan 13;20:20. doi: 10.1186/s12870-019-2227-7 (PMC6958797; doi:10.1186/s12870-019-2227-7)
Supplement: Supplementary file 6 — Additional file 6. Divergence between paralogous SsHAK gene pairs in Saccharum spontaneum. [file 12870_2019_2227_MOESM6_ESM.docx]

**Additional file 6:** Divergence between paralogous *SsHAK* gene pairs in *Saccharum spontaneum*.

| Gene 1 | Gene 2 | Ka | Ks | Ka/Ks | Divergence  time (Mya) | *p*-value |
| --- | --- | --- | --- | --- | --- | --- |
| *SsHAK5a* | *SsHAK5b* | 0.076 | 0.231 | 0.327 | 18.94 | 7.63E-14 |
| *SsHAK16a* | *SsHAK16b* | 0.093 | 0.709 | 0.131 | 58.14 | 6.24E-50 |
