# Supplementary material for: Genome-wide systematic characterization of the HAK/KUP/KT gene family and its expression profile during plant growth and in response to low-K+ stress in Saccharum
Source: BMC Plant Biol. 2020 Jan 13;20:20. doi: 10.1186/s12870-019-2227-7 (PMC6958797; doi:10.1186/s12870-019-2227-7)
Supplement: Supplementary file 8 — Additional file 8. The proportion of different numbers of exons in all HAKs from 15 plant species. [file 12870_2019_2227_MOESM8_ESM.docx]

**Additional file 8** The proportion of different number of exons in all *HAKs* from 15 plant species

| Exon number | Gene number | Proportion |
| --- | --- | --- |
| 2 | 4 | 1.43% |
| 3 | 6 | 2.15% |
| 4 | 8 | 2.87% |
| 5 | 3 | 1.08% |
| 6 | 14 | 5.02% |
| 7 | 19 | 6.81% |
| 8 | 49 | 17.56% |
| 9 | 125 | 44.80% |
| 10 | 43 | 15.41% |
| 11 | 4 | 1.43% |
| 12 | 3 | 1.08% |
| 16 | 1 | 0.36% |
