# Supplementary material for: Genome-wide systematic characterization of the HAK/KUP/KT gene family and its expression profile during plant growth and in response to low-K+ stress in Saccharum
Source: BMC Plant Biol. 2020 Jan 13;20:20. doi: 10.1186/s12870-019-2227-7 (PMC6958797; doi:10.1186/s12870-019-2227-7)
Supplement: Supplementary file 10 — Additional file 10. The primers for the RT-qPCR verification of four HAK genes in Saccharum hybrid YT55. [file 12870_2019_2227_MOESM10_ESM.docx]

**Additional file 10:** The primers for RT-qPCR verification of four *HAK* genes in *Saccharum* hybrid YT55.

| Gene name | Forward primer(5’-3’) | Reverse primer(5’-3’) |
| --- | --- | --- |
| *HAK1* | TGCTCTTCACCCTCACAATC | CTTCTCCTTGATCCCGCTTAC |
| *HAK7* | GTGTGGTGTATGGAGACTTGAG | CAGAGAAAGCAAGCCAAAGATG |
| *HAK20b* | TCTCACCCAACCTCCCATAA | CTGCAGCTCCCTCTGAATAAA |
