# Supplementary material for: Genome-wide systematic characterization of the HAK/KUP/KT gene family and its expression profile during plant growth and in response to low-K+ stress in Saccharum
Source: BMC Plant Biol. 2020 Jan 13;20:20. doi: 10.1186/s12870-019-2227-7 (PMC6958797; doi:10.1186/s12870-019-2227-7)
Supplement: Supplementary file 11 — Additional file 11. The primers used to clone SsHAK1 and SsHAK21 and construct the yeast expression vector. [file 12870_2019_2227_MOESM11_ESM.docx]

**Additional file 11:** The primers used for *HAK1* and *HAK21* clone and constructing yeast expression vector

| Primer name | Sequence of primer (5’-3’) |
| --- | --- |
| HAK1-F (in-fusion) | CAGTGTGGTGGAATTCCATGTCGTCGTCGCTGGAG |
| HAK1-R (in-fusion) | CGAAGGGCCCTCTAGCTATATTTCATATGTGATGCCAACC |
| HAK21-F (in-fusion) | CAGTGTGGTGGAATTCCATGGTTCTCACTGCCGAG |
| HAK21-R (in-fusion) | CGAAGGGCCCTCTAGCTAGATTTCATATGAGAT |
